# Deconstructing the effects of stochasticity on transmission of hospital-acquired infections in ICUs

**DOI:** 10.1098/rsos.230277

**Published:** 2023-09-13

**Authors:** Fardad Haghpanah, Gary Lin, Eili Klein, for the CDC MInD-Healthcare Program

**Affiliations:** ^1^ One Health Trust, Washington, DC, USA; ^2^ Department of Emergency Medicine, Johns Hopkins School of Medicine, Baltimore, MD, USA

**Keywords:** hospital-acquired infection, intervention, seasonality, agent-based modelling, simulation

## Abstract

The inherent stochasticity in transmission of hospital-acquired infections (HAIs) has complicated our understanding of transmission pathways. It is particularly difficult to detect the impact of changes in the environment on acquisition rate due to stochasticity. In this study, we investigated the impact of uncertainty (epistemic and aleatory) on nosocomial transmission of HAIs by evaluating the effects of stochasticity on the detectability of seasonality of admission prevalence. For doing so, we developed an agent-based model of an ICU and simulated the acquisition of HAIs considering the uncertainties in the behaviour of the healthcare workers (HCWs) and transmission of pathogens between patients, HCWs, and the environment. Our results show that stochasticity in HAI transmission weakens our ability to detect the effects of a change, such as seasonality patterns, on acquisition rate, particularly when transmission is a low-probability event. In addition, our findings demonstrate that data compilation can address this issue, while the amount of required data depends on the size of the said change and the degree of uncertainty. Our methodology can be used as a framework to assess the impact of interventions and provide decision-makers with insight about the minimum required size and target of interventions in a healthcare facility.

## 1. Introduction

Hospital-acquired infections (HAIs) pose a significant threat to patient safety and are a major burden on the healthcare system. Approximately 4% of hospitalized patients in the United States acquire an HAI during their stay, resulting in more than 1.7 million HAIs annually in the USA [1]. HAIs prolong hospital stays and increase rates of mortality, with the estimated annual cost of the most common HAIs to the US healthcare system ranging from $8 to $12 billion [2]. The emergence of multidrug-resistant organisms (MDROs) has further magnified the challenge of HAIs by increasing the complexity and cost of treatment [3]. Despite the significant costs and negative consequences for patients from HAIs, uncertainty remains regarding the pathways of transmission of HAI-causing organisms. Although it is widely believed that organisms spread in hospitals due to poor infection control practices by healthcare workers (HCWs), e.g. non-compliance with handwashing, the complexity of HCW–patient networks, constant movement of HCWs, and frequent contact with surfaces and devices make nosocomial transmission pathways for HAIs a challenge to investigate.

The uncertainty regarding transmission of HAI-causing organisms can be categorized into two types: (1) *epistemic uncertainty*, which refers to the uncertainty due to lack of knowledge or noise in data, and (2) *aleatory uncertainty*, which refers to the inherent variability in certain parameters. Epistemic uncertainty can potentially be reduced by collecting more data and improving the accuracy of data collection, e.g. improving estimates of prevalence of colonization on admission. However, aleatory uncertainty is intrinsic to the nature of events, e.g. length of stay or HCW hygiene compliance. Aleatory uncertainty is more commonly referred to as *stochasticity*, and increased data collection cannot reduce the inherent uncertainty in these variables. For clarity, hereinafter, we refer to aleatory uncertainty as ‘stochasticity’, and we use ‘uncertainty’ when referring to randomness, in general.

Uncertainty analysis techniques have been developed to analyse the impact of uncertainties on system outcomes in parameters for which the true values are not known (i.e. epistemic uncertainty) [4,5], while stochastic modelling techniques, such as agent-based modelling (ABM) and Markov chains, have been used to account for stochasticity in systems [6]. Accounting for stochasticity in the study of complex systems is crucial to help understand the behaviour of such systems under different circumstances, such as environmental or operational changes or interventions. Furthermore, dynamical effects of stochasticity may result in variations to a deterministic response such that deterministic models would be incapable of explaining the data. For example, Rohani *et al*. [7] showed that stochasticity could have surprising impacts on the dynamics of whooping cough and measles—two common infectious diseases with relatively similar natural history and reproductive potentials. Their analyses showed that due to the differences in the epidemiological time scales of these two diseases, whooping cough epidemics could be strongly stochastic such that deterministic approaches may fail to explain the observed incidence data, while measles could be well explained using deterministic models as its dynamics are less sensitive to the variability in the infectious period.

In addition, simulations have shown that inherent stochasticity can cause dynamics similar to what external deterministic forces can cause. For example, when considering spatial correlations in epidemics, demographic stochasticity has been shown to be able to cause both small post-outbreak waves and major secondary outbreaks. This implies that in highly connected contact networks, recurrent epidemics can be due to endogenous stochasticity, rather than exogenous factors, such as seasonality or introduction of new variants [8,9].

One area that needs further investigation, particularly in the context of HAI transmission, is the effect of stochasticity on the detectability (i.e. inferability using data analysis techniques) of underlying deterministic trends (e.g. long-term continuous increase in antibiotic consumption leading to an increase in colonization susceptibility) and epistemic variabilities, such as seasonality in admission prevalence or the long-term impact of infection prevention and control interventions. Here, admission prevalence refers to the proportion of patients who are already colonized when admitted to ICUs (i.e. pre-admission colonization). For example, the impact of seasonality on the outcome may diminish when the amplitude of stochasticity increases, such that for a highly stochastic process, it may become difficult to detect the seasonal component (see electronic supplementary material, figure S1). These issues are compounded when the event is a relatively low-probability event, such as an HAI.

Data compilation (i.e. compiling data from multiple sources) is a common approach when dealing with highly stochastic events. While collecting more and better data generally does not reduce aleatory uncertainty, with highly stochastic events, such as acquisition of HAIs, compiling data from multiple sources can help to detect underlying deterministic patterns, such as seasonality, that may be concealed by strong stochasticity (electronic supplementary material, figure S2). Although data compilation can be a solution, given such data are available, certain precautions about spatio-temporal heterogeneities in the data have to be considered, particularly when studying the dynamics of disease transmission [10].

One of the main principles of systems engineering is the necessity of understanding the underlying structure and dynamics of a process if one wants to implement effective changes. When it comes to complex processes such as nosocomial transmission of HAIs, this means we need to understand the underlying deterministic and stochastic drivers of pathogen transmission, and their interconnections, before we can decide what intervention would be effective and estimate or measure its impact after implementation. We need to quantify the effects of seasonality and possible trends in admission prevalence, which is arguably one of the main drivers of nosocomial transmission of HAIs. In simple words, how can we measure the impact of interventions if we do not know how many (or what proportion of) patients are already colonized when they are admitted to ICUs, and how this may change through time (i.e. seasonality patterns) and affect the effectiveness of interventions. If we see a decrease in infection rate, we need to know whether it has been due to an intervention, the effects of seasonality, or just randomness. Accordingly, investigation of the seasonality patterns in the drivers of nosocomial transmission of HAIs precedes the analysis of intervention impacts.

In this study, we investigated the impact of sources of uncertainty (epistemic and aleatory) on nosocomial transmission of HAIs. Specifically, we evaluated the effect of stochasticity on the propagation of seasonality from admission to acquisition. We demonstrate how varying levels of stochasticity and seasonality could collectively affect the acquisition rate. Additionally, we demonstrate the amount of data needed to overcome the stochasticity effects and detect underlying deterministic patterns, which can improve our understanding of transmission pathways and would provide insight on targeted and more effective interventions to mitigate acquisition.

## 2. Methods

We developed an agent-based model of an ICU to study the effects of uncertainties on the dynamics of transmission of HAIs. We simulated the transmission of HAI-causing pathogens under different seasonality and admission prevalence scenarios, considering the uncertainties in the behaviour of the healthcare workers (HCWs) and stochasticity of the transmission of pathogens between patients, HCWs, and the environment. Finally, we trained a logistic regression to infer the parameter space in which seasonality effects is likely to be observed.

### 2.1. The agent-based model

#### 2.1.1. Agents

We explicitly defined two types of human agents: patients and HCWs. HCW agents are further divided into two categories of nurses and physicians. Other HCWs, such as health technicians and nonclinical staff, were not included in the model as nurses and physicians constitute the majority of patient visits [11]. Patient rooms in the ICU were also modelled as agents. Visitors were not included.

In the model, patients can be in one of four disease states: susceptible to colonization (*S*), highly susceptible to colonization due to antibiotic use (*X*), colonized (*C*) and infected (*I*). Upon admission, each patient's disease state is assigned using a multinomial trial with defined probabilities for admission status distribution. To address the role of infection prevention, infected patients and those identified as colonized are put on contact precautions, which affects the interaction with HCWs and transmission potential (see Process overview).

Each patient stays in a single room and has a primary nurse and a primary physician. Nurses in the ICU are only assigned to ICU patients. Physicians can potentially visit patients from multiple units in the hospital; however, as there is only one unit simulated in this study, this feature of the model is not used and is reserved for future expansions of the study.

Patient rooms are modelled as single-bed rooms. A history of all patient movement in/out/between rooms is maintained. Rooms can become contaminated by the residing patient or visiting HCWs. Room contamination is characterized using a binary variable. Other spatial characteristics of the ICU, e.g. room dimensions and corridors, were not modelled.

#### 2.1.2. Process overview

##### 2.1.2.1. Simulation setup

At the beginning of each simulation, the hospital is initialized by creating one ICU with a pre-determined patient capacity and the physicians as their primary working area is the entire hospital. The number of physicians is determined based on the doctor-to-patient ratio and the capacity of the ICU. Upon initializing the ICU, the patient rooms are created based on the ICU capacity, and the ICU nurses are created based on the nurse-to-patient ratio. Next, an initial group of patients is created. Each patient gets assigned to the least busy nurse and physician. If more than one nurse (or physician) has the same number of patients, the new patient is randomly assigned to one of the nurses (or physicians). The patient is then randomly assigned to an empty room, and its length of stay is randomly determined from a lognormal distribution. At admission patients are randomly selected for pathogen testing, and given the test accuracy (sensitivity), colonized patients may be identified and put under contact precautions. Contact precautions, as a type of transmission-based precautions, are a set of second tier infection control measures for patients with known (or suspected) infectious agents. Patients under contact precautions are placed in single-patient rooms, have limited movements and visits for medically necessary purposes only, and must use disposable or dedicated patient-care equipment. For all interactions that may involve contact with the patient or the patient's environment, personal protective equipment (PPE), such as gloves and gown, must be used [12].

##### 2.1.2.2. Daily initialization procedure

At the start of every day, patients that have completed their stay are discharged. After a patient is discharged, the terminal disinfection protocol is executed in the room, which can clear the room contamination based on the room disinfection probability. Second, based on the bed utility rate, sampled for each empty bed (i.e. room), the number of admitted patients for the day is calculated, and new patients are initialized and assigned to their primary HCWs and rooms, as explained in setup. Afterward, the nurses' (and physicians’) daily visit schedules are determined by generating a random number of visits that each patient receives from nurses (and physicians) during a day (24 h). Patients under contact precautions receive fewer visits from the HCWs [13]. While each patient has a primary nurse, there may be a few random visits by other nurses. Accordingly, based on the primary nurse visit rate, a proportion of these visits are assigned to the primary nurse, where the visits take place randomly at different times during the day. The remaining nurse visits are randomly assigned to other nurses. Physician visits are assigned similarly, except that all visits for a patient are from a single primary physician.

##### 2.1.2.3. Daily routine

**Figure 1.**
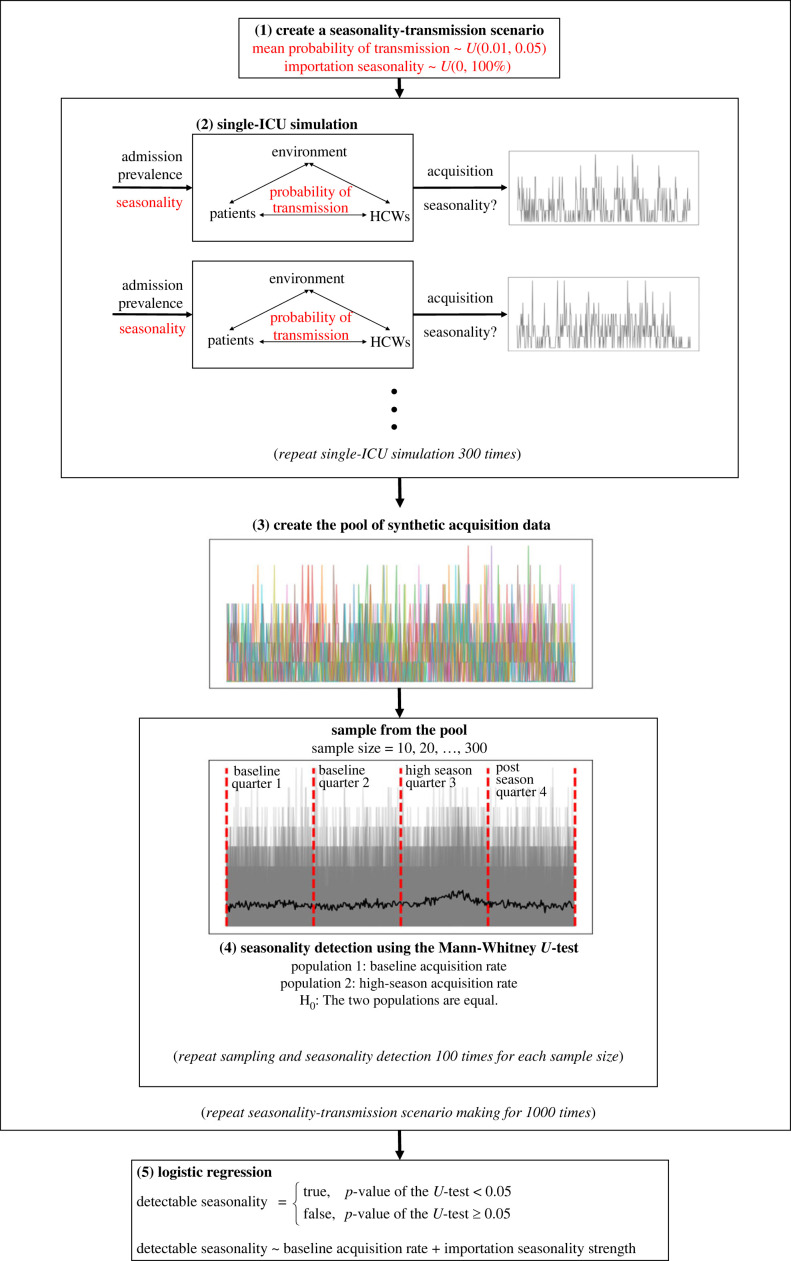
Schematic representation of the research methodology: (1) sampling seasonality and transmission parameters to create 1000 scenarios; (2) performing single ICU simulations for 300 times for each seasonality-transmission scenario (with other parameters sampled per each simulation); (3) compiling the results of 300 000 simulations including the values of sampled parameters into a pool of synthetic acquisition data; (4) examining seasonality detection rate using the Mann–Whitney *U* test for each seasonality-transmission scenario by incrementally increasing the size of samples drawn from our data pool; (5) training a logistic regression to identify the parameter space (seasonality strength, mean probability of transmission, and sample size) for which seasonality is statistically more likely to be detectable.

The simulation is run for 360 days with a one-hour time step. The simulation is segmented into four 90-day quarters where the third quarter is the high season during which admission prevalence is higher (figure 1). Every hour, the nurses and physicians conduct their scheduled visits to the patients. During each visit, the HCW wears PPE if the patient is under contact precautions. Therefore, a contaminated HCW wearing PPE will not transmit to the patient, but the HCW will remain contaminated. If the patient is not under contact precautions, the HCW may wash their hands, given the nurse's hand hygiene compliance probability on entry. If the HCW complies, hand washing may clear any possible contamination based on the hand washing efficacy rate. Before the HCW comes in contact with the patient, they will interact with the environment of the patient's room. This allows for both environmental shedding by contaminated HCWs and HCW contamination from a contaminated environment. If the HCW is wearing PPE, this will prevent environmental shedding, but the PPE can become contaminated when coming in contact with a contaminated environment. This increases the risk of transmission for the patient; however, since the HCWs discard PPE after each visit, the risk of transmission for the next patient will remain unchanged.

During contacts with the patient, if the HCW or their PPE is contaminated, the pathogens may transmit and colonize the patient. Similarly, a colonized patient can contaminate the HCW or their PPE. It is assumed that after contacting the patient, the HCW contacts the environment again, which may contaminate the environment itself. However, if the PPE becomes contaminated before the HCW leaves the room, this will not increase the risk of transmission as the PPE will be discarded. The HCW may comply with hand washing when exiting the room, based on the hygiene compliance probability on exit. Environmental contamination and patient colonization occur based on probability of environmental contamination by a contaminated HCW $\left(\beta_{\mathrm{he}}\right)$, probability of HCW contamination from a contaminated surface $\left(\beta_{\mathrm{eh}}\right)$, probability of patient colonization from a contaminated HCW $\left(\beta_{\mathrm{hp}}\right)$, and probability of HCW contamination from a colonized patient $\left(\beta_{\mathrm{ph}}\right)$. It is assumed that $\beta_{\mathrm{he}}=\beta_{\mathrm{eh}}=\beta_{\mathrm{ph}}=2\beta_{\mathrm{hp}}$ [14]. To account for direct environmental transmission in a contaminated room, a Bernoulli trial is conducted every hour with a success probability equal to 1/24 (one twenty-fourth) of $\beta_{\mathrm{hp}}$, i.e. the probability of direct environmental transmission for each patient for 24 h is the same as the probability of transmission from a contaminated HCW during one visit. HCWs' interactions were not modelled due to lack of evidence for the contribution of such interactions to transmission [15].

##### 2.1.2.4. Infections

A patient's colonization status may deteriorate into an infection based on a probability of infection from colonization. This is implemented using a Bernoulli trial every day with a success probability equal to the probability of infection. When a patient becomes infected, they will be put under contact precautions and a 7-day course of antibiotic treatment, which may extend the patient's length of stay. The antibiotic treatment period may extend over seven days until the patient clears the infection with a negative culture test. After clearing the infection, the patient will be removed from contact precautions.

##### 2.1.2.5. Active surveillance

Active surveillance is conducted once every 7 days on selected patients starting from day 1 in every simulation. Given the test accuracy (sensitivity), if a patient's test is positive, the patient will be put under contact precautions for the rest of their stay.

#### 2.1.3. Design concepts

The model includes HCW-mediated and environmental transmission during HCW visits. HCW-to-HCW transmission was not included. Patient-to-patient transmission was not considered as patients are highly unlikely to have direct interactions with other patients in ICUs. We assumed that it is more likely for a HCW to become contaminated from a colonized patient than for a susceptible patient to become colonized from a contaminated HCW, as it is easier for pathogens to transfer to hands (of a HCW) than to establish within the microbiome of a patient and cause colonization [14]. We assumed the HCWs work multiple shifts per day. For the sake of computational performance, when a HCW's shift is over, the contamination status of the HCW is removed to account for the starting shift of a new HCW, instead of creating a new HCW agent.

Patients that have been or are receiving antibiotics have been shown to be more susceptible to colonization, particularly with MRSA [16,17]. The risk of colonization for more susceptible patients is modified by an increase factor π>1, so the probability of colonization from a contaminated HCW for more susceptible patients is $\pi \beta_{\mathrm{hp}}$.

### 2.2. Parameterization

We defined the constant parameters and the statistics of random variables of the model based on the findings from the literature. While the mean probability of transmission and seasonality are assumed not to vary across ICUs, other parameters can vary for different ICUs, the probabilities of which are specified in table 1. We have represented parameter uncertainty with uniform distributions as they allow random parameters to vary equally within reasonable bounds.

**Table 1.** Summary of the model parameters and their probability distributions or values.

parameter	distribution/value	source	sampled per
capacity	25	assumed	—
physician-to-patient ratio	0.1	per [18]	—
nurse-to-patient ratio	0.5	per [18]	—
initial population	20	assumed	—
bed utilization rate	uniform (*a* = 0.55, *b* = 0.9)	[19,20]	day
highly susceptible ratio $\left(P_{X}/P_{S}\right)$	uniform (*a* = 0.5, *b* = 0.9)	assumed, based on [21]	simulation
admission prevalence (colonization importation ratio)	uniform (*a* = 0.01, *b* = 0.15)	assumed	simulation
infection importation ratio	0	assumed	—
length of stay	lognormal (μ = 0.693, σ = 1.058)	[21,22]	patient
patient testing rate on admission	100%	assumed	—
patient testing accuracy (sensitivity)	80%	assumed	—
nurses’ shift length	8 h	assumed	—
primary nurse visit rate	uniform (*a* = 0.5, *b* = 1.0)	assumed, based on [23]	simulation
physician hygiene compliance probability on entry	uniform (*a* = 0.3, *b* = 0.5)	[23–26]	physician
physician hygiene compliance probability on exit	uniform (*a* = 0.4, *b* = 0.7)	[23–26]	physician
nurse hygiene compliance probability on entry	uniform (*a* = 0.5, *b* = 0.7)	[23,25]	physician
nurse hygiene compliance probability on exit	uniform (*a* = 0.6, *b* = 0.9)	[23,25]	nurse
physician PPE compliance probability	uniform (*a* = 0.7, *b* = 0.9)	[23,27]	nurse
nurse PPE compliance probability	uniform (*a* = 0.8, *b* = 0.9)	[23,27]	nurse
physician contacts per patient per day	uniform (*a* = 5, *b* = 10)	[13,23]	day
physician contacts per patient per day (under contact precautions)	uniform (*a* = 3, *b* = 6)	[13,23]	day
nurse contacts per patient per day	uniform (*a* = 20, *b* = 39)	[13,23]	day
nurse contacts per patient per day (under contact precautions)	uniform (*a* = 13, *b* = 26)	[13,23]	day
hand hygiene efficacy	uniform (*a* = 0.7, *b* = 0.99)	[28]	simulation
terminal room disinfection efficacy	uniform (*a* = 0.4, *b* = 0.6)	[25]	simulation
infection treatment length	7 days	[29,30]	—
transmission increase factor for highly susceptible	uniform (*a* = 1.5, *b* = 3.5)	[31,32]	simulation
mean probability of room contamination by colonized patients per day	uniform (*a* = 0.01, *b* = 0.05)	assumed, based on [14]	simulation
mean probability of transmission from contaminated HCW to susceptible patient	uniform (*a* = 0.005, *b* = 0.025)	assumed, based on [14]	simulation
mean probability of transmission from colonized patient to uncontaminated HCW	uniform (*a* = 0.01, *b* = 0.05)	assumed, based on [14]	simulation
mean probability of HCW contamination from contaminated environment	uniform (*a* = 0.01, *b* = 0.05)	assumed, based on [14]	simulation
mean probability of environmental contamination from contaminated HCW	uniform (*a* = 0.01, *b* = 0.05)	assumed, based on [14]	simulation
probability of natural progression from colonization to infection	uniform (*a* = 0, *b* = 0.02)	assumed	simulation
probability of direct infection from exposure	uniform (*a* = 0, *b* = 0.05)	assumed	simulation
probability of patient direct environmental colonization	uniform (*a* = 0, *b* = 0.05)	assumed	simulation
shedding increase factor for infected patients	uniform (*a* = 1, *b* = 2)	assumed, based on [14]	simulation
pathogen natural clearance rate from dry surfaces per day	uniform (*a* = 0, *b* = 0.01)	[33,34]	simulation
seasonality strength in admission prevalence	uniform (*a* = 0%, *b* = 100%)	assumed	simulation

The event probabilities for the multinomial distribution of admission status are established based on the highly susceptible (*P_X_*) and colonization importation (*P_C_*) ratios. After sampling *P_C_* and *P_X_* from their respective distributions, the probability of admission as susceptible, assuming no infection importation $\left(P_{I}=0\right)$, can be calculated as: $P_{S}=1-P_{X}-P_{C}$. Given all event probabilities, the admission status of each patient is determined using a multinomial trial, defined as *Multinomial (1, {P_S_, P_X_, P_C_, P_I_})*.

### 2.3. Stochasticity and seasonality measures

The parameters that control the direct transmission (i.e. force of infection) during a HCW–patient contact are the probability of environmental contamination by a colonized/infected patient, the probability of transmission from a contaminated HCW to a susceptible patient, the probability of transmission from a colonized/infected patient to an uncontaminated HCW, the probability of HCW contamination from the environment, and the probability of environmental contamination from a contaminated HCW. Other parameters, such as hand hygiene, PPE compliance, and number of visits per day, affect the overall probability of transmission through time. In total, the random variables of the model, represented using distributions listed in table 1, constitute the stochastic behaviour of the model. Given that the objective of the simulations is to evaluate the impact of stochasticity on seasonality effects, acquisition rate, expressed as the number of acquisitions per 1000 patient-days, is calculated and reported as the main outcome of interest, which essentially measures the overall likelihood of transmission.

We modelled the seasonality effect as an increase in admission prevalence of colonization during the high-season period using a Gaussian-modulated sinusoidal function to generate a Gaussian pulse:

$$P_{C}^{\mathrm{hs}}=P_{C}^{b}+\Sigma e^{-1/2\sigma^{2}{\left(t-t_{c}\right)}^{2}},$$

where $P_{C}^{\mathrm{hs}}$ is the admission prevalence during the high season, $P_{C}^{b}$ is the average admission prevalence during the baseline period, Σ is the seasonality strength, *t_c_* is the peak day of the high season (assuming the symmetrical shape of the Gaussian pulse), and σ is the pulse relative half-duration, which corresponds to the standard deviation of the unit pulse ($T_{p}=2\pi \sigma \to \sigma =90/2\pi =14.324$ days). Seasonality strength is expressed in percentages, e.g. a 50% seasonality strength implies that the peak of admission prevalence during the high season is 50% higher than that of the baseline.

The seasonality impact on quarterly acquisition rate (i.e. significance of seasonality) is assessed using the Mann–Whitney *U*-test [35]. Since the distribution of acquisition rate is highly right-skewed (figure 3), the non-parametric *U*-test provides a more robust assessment than the Student's *t*-test typically used for hypothesis testing.

There is a potential relationship between the detectability of seasonality in acquisition rate, the seasonality strength of admission prevalence, and the baseline acquisition rate. However, because of the uncertainties in transmission, this relationship is not deterministic, so the *U*-test may or may not show seasonality in acquisition rate across simulations with similar parameters. As the amplitude of the seasonal signal in admission prevalence and the baseline acquisition rate increase, seasonality effect in acquisition rate is magnified and is more likely to be detected. We used a logistic regression predictor to define the parameter space for which the seasonality effect is most likely to be detected. The logistic regression predictor is trained with seasonality strength of admission prevalence and baseline acquisition rate as the predictor variables and the binary outcome of the *U*-test as the response variable.

### 2.4. Simulations

The main outcome of interest is quarterly acquisition rates, in terms of number of acquisitions per 1000 patient-days. To evaluate the combined effect of uncertainty in transmission and seasonality on acquisition rate, 1000 transmission-seasonality scenarios were created by sampling the mean probability of transmission and seasonality strength from their respective distributions using Latin hypercube sampling (LHS), assuming that mean probability of transmission and seasonality strength do not vary across ICUs. For each scenario, a single ICU was simulated, and the simulation was repeated 300 times using Monte Carlo methods. The number of simulation repetitions (i.e. 300) was determined by sampling error minimization, which showed that the distribution statistics (sample mean and standard deviation) converged when sample size was 300 or larger (electronic supplementary material, figure S3). In other words, repeating ICU simulation for each of the transmission-seasonality scenarios for more than 300 times does not add more information to the mean and variance of model outputs, i.e. acquisition and infection rates. Each ICU was simulated for 360 days after a 60-day burn-in period. An overview of the methodology is illustrated in figure 1.

## 3. Results

### 3.1. Baseline results and validation

**Figure 2.**
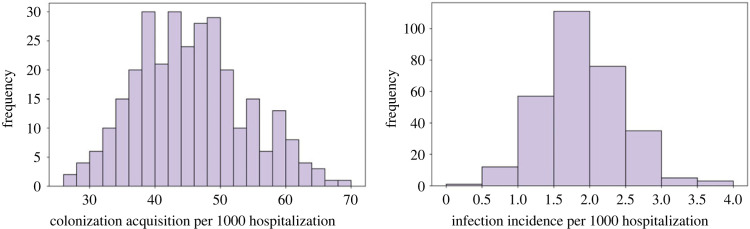
Histogram of acquisition (colonization) and infection rates at baseline (i.e. no seasonality effects) from 300 simulations.

At baseline, with no seasonality effect, the mean acquisition rate was 45.55 (SD, 8.35) cases per 1000 patient-days. The 95% highest density interval (HDI) of acquisition rate extends from 30.55 to 62.05 cases, which agrees with the literature [23,36,37]. These statistics translate into an acquisition risk of 0 to 12.5% with an average of 9% per ICU hospitalization. Mean infection rate was 1.90 (SD, 0.57) cases per 1000 patient-days (HDI: 0.76–2.76). Infection risk was 0 to 0.6% with an average of 0.4% per ICU hospitalization (figure 2).

### 3.2. Simulation results

**Figure 3.**
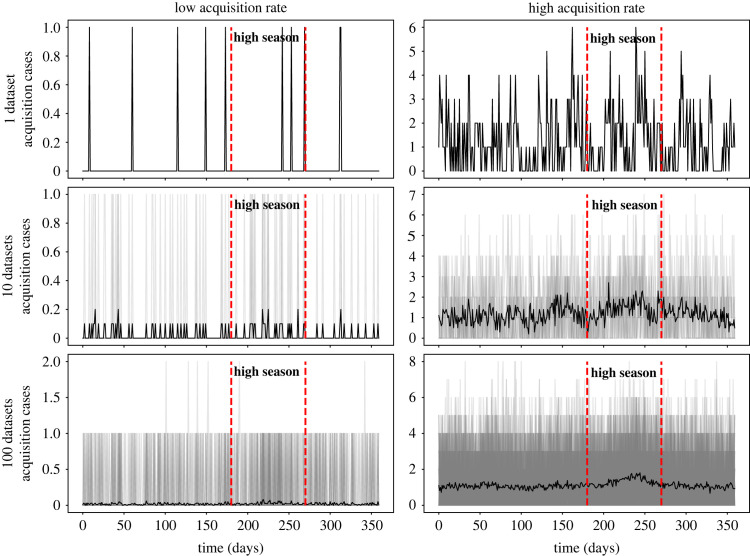
Effect of acquisition probability on the detectability of seasonality effects. The left column is a scenario in which admission prevalence and mean probability of transmission are relatively low (0–5% and 1%, respectively), and the right column is an example of a scenario with relatively high admission prevalence and mean probability of transmission (5–10% and 8%, respectively). In both scenarios, seasonality strength is 100%. While adding more data (i.e. running more simulations) helps with identifying the seasonal pattern in the right column where acquisition is more likely, no seasonality effects can be detected in the left column where baseline acquisition rate is low.

The effect of importation seasonality on the acquisition rate depends on the likelihood of transmission. Importation seasonality may not have a significant effect on incidence when transmission is a low-probability event (figure 3).

**Figure 4.**
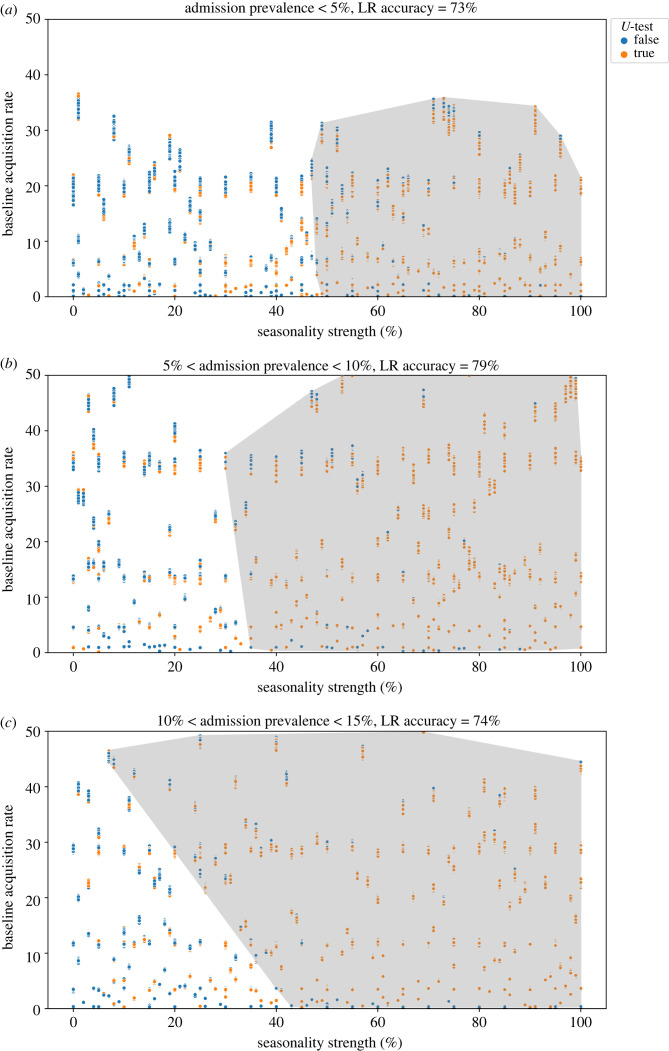
Detectability of seasonality effects as a function of baseline acquisition rate (cases per 1000 patient-days) and seasonality strength of admission prevalence. Each data point represents a 365-day ICU simulation with the corresponding seasonality strength and resulting baseline quarterly acquisition rate, where the green circles represent those simulations for which the seasonality effect was detectable during the high season using the *U*-test, and the blue ones are simulations in which seasonality was undetectable. The shaded area shows the results of the logistic regression predictor (the parameter space corresponding to the ‘true’ seasonality detection predictor).

To better investigate the patterns in the effects of seasonality and stochasticity on acquisition rate, first we categorized the simulations by the admission prevalence range, i.e. 0–5%, 5–10% and 10–15% (figure 4). For comparable values of seasonality strength and acquisition rate, when admission prevalence is higher, the effect of seasonality on acquisition rate increases. When admission prevalence is less than 5%, seasonality effects could only be detected when the seasonality strength was more than 50%, regardless of the acquisition rate value (figure 4*a*). For admission prevalence between 5 and 10%, seasonality effects could only be detected when the seasonality strength was more than 40% (figure 4*b*). Similar patterns were observed for higher admission prevalence values (10 to 15%). Seasonality effects were detectable when the seasonality strength was more than 25% and acquisition rate was more than 30 cases per 1000 patient-days. As acquisition rate decreased, the minimum detectable seasonality strength increased such that for acquisition rates below 5 cases per 1000 patient-days, the minimum detectable seasonality strength was 40% (figure 4*c*). The accuracy of the logistic regression predictor of the detectable seasonality region (highlighted in figure 4) was 72–79%. In other words, when the seasonality strength and baseline acquisition rate fall within the predictable seasonality region, there is still an approximate 20–30% chance that the seasonality effect will not be detected in the data. The reason is the effects of stochasticity which add noise to the observed acquisition rate and weaken the seasonality signal in the data.

**Figure 5.**
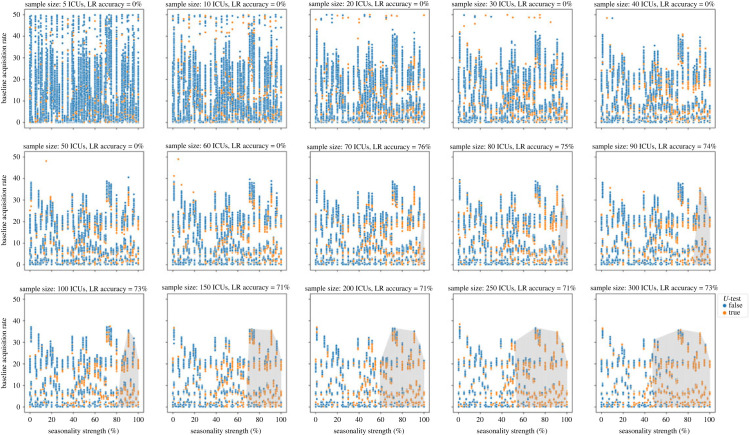
Effect of sample size on seasonality detectability as a function of baseline acquisition rate (cases per 1000 patient-days) and seasonality strength, when admission prevalence is lower than 5%. Green circles represent simulations for which the seasonality effect was detectable during the high season using the *U*-test, and the blue ones are simulations in which seasonality was undetectable. The shaded area shows the results of the logistic regression predictor (the parameter space corresponding to the ‘true’ seasonality detection predictor).

The effect of sample size on seasonality detection for admission prevalence of less than 5% is shown in figure 5 (see electronic supplementary material, figures S4 and S5, for the results for higher admission prevalence ranges). In general, as the sample size increased, the detectable seasonality region expanded to smaller seasonality strengths and acquisition rates. When admission prevalence was less than 5%, the detectable seasonality region only appears when at least 80 ICU simulations were used in the *U*-test, i.e. for sample sizes of smaller than 80 ICUs, no specific pattern was identified in the parameters of interest between the seasonality-detectable and undetectable simulations. The detectable seasonality region did not change for sample sizes of 250 and larger. The accuracy of the seasonality detection predictor ranges from 69 to 75%.

For admission prevalence of 5–10%, the minimum sample size for seasonality detection was 50 ICUs, while this was only limited to seasonality strength of 90% and more. The detectable seasonality region did not change for sample sizes of 250 and larger. The accuracy of the seasonality detection predictor was 71–79%. For simulations with an admission prevalence of more than 10%, similar patterns were observed.

Overall, when seasonality strength was less than 25%, no statistically significant relationship was found between acquisition rate, seasonality strength, and seasonality effect detectability, regardless of sample size and the level of acquisition rate. For seasonality strength of 20–40%, the seasonality effect was only statistically detectable when admission prevalence was higher than 10% and sample size was larger than 200 ICUs and acquisition rate was higher than 5 cases per 1000 patient-days.

**Figure 6.**
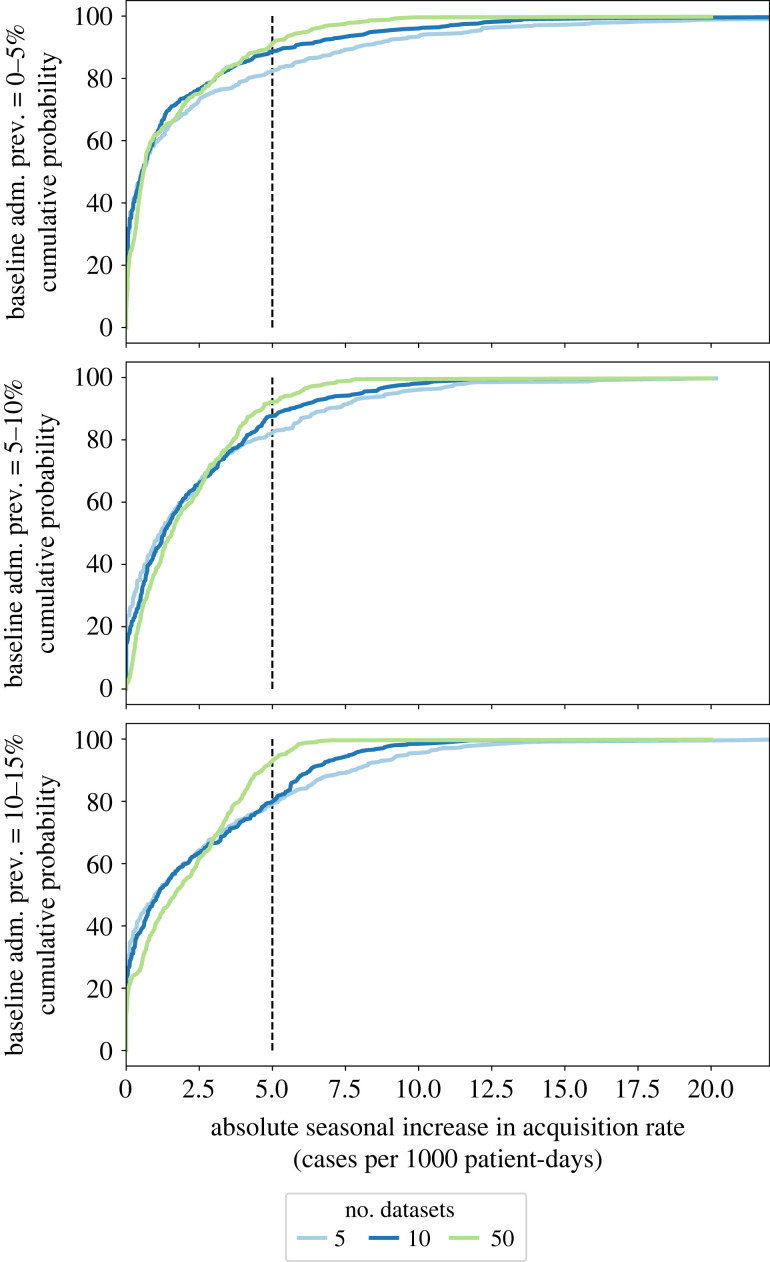
Cumulative probability distribution of the absolute increase in acquisition rate due to a 100% seasonality in admission prevalence (i.e. a 100% increase in admission prevalence during the peak of the high season with respect to the baseline), for different sample sizes (number of ICUs).

The effects of sample size on seasonality detectability can also be studied based on the law of small numbers [38]. For small sample sizes, the distribution of the consequent increase in acquisition rate due to seasonality in admission prevalence has a higher variance. For example, when baseline admission prevalence was 10–15%, a 100% seasonality strength was associated with an average of 2.57 cases (per 1000 patient-days) increase in acquisition rate and a standard deviation of 3.45 cases, when the sample included only 5 ICUs. When 50 ICUs were considered, the mean and standard deviation were 2.06 and 1.96 cases, and for 250 ICUs, the statistics were 2.04 and 1.72 cases. Looking at the cumulative probability distribution functions (CDFs) of the seasonal increase in acquisition rate due to a 100% seasonality strength in admission prevalence (figure 6), the probability of an increase of more than 5 cases was 83 to 91% when baseline admission prevalence rate was less than 5%, for different sample sizes. However, when admission prevalence rate was 10–15%, the probability of an increase of more than 5 cases was 20%, 7%, and less than 1% when sample size was 5, 50 and 250 ICUs, respectively.

### 3.3. Uncertainty analysis

We used partial rank correlation coefficients (PRCC) to evaluate the contribution of variability of input parameters to the variability of outcome variables. The results show that the stochasticity in acquisition rate is mainly due to the stochasticity in admission prevalence, probability of transmission between HCWs and patients, and probability of environmental contamination or colonization by HCWs and patients. While for infection rate, admission prevalence and the probability of progression (from colonization to infection) mainly contributed to stochasticity. Refer to the supplementary materials for details.

## 4. Discussion

The distinction between epistemic and aleatory (stochasticity) uncertainties has important implications in understanding the transmission of HAIs, as it helps to identify the sources of uncertainty which are potentially reducible. Our simulations demonstrate that the inherent stochasticity in HAI transmission weakens our ability to detect the effects of deterministic forces, such as seasonality, on the acquisition rate. From a broader perspective, the amount of stochasticity is a critical driver in our ability to detect the impact of an intervention on reducing the acquisition rate from baseline. Here, we used seasonality in admission prevalence as an example of a (short-term) deterministic change to the dynamics; however, our methodology and findings are intended to represent a framework for evaluating the ability to detect the impact of interventions intended to alter the acquisition rate of HAIs.

The core message of our findings is that due to the highly stochastic nature of HAI transmission, the impact of changes in the environment (whether short-term seasonality effects or long-term interventions) may not be readily detectable unless the size of the change is larger than a certain threshold (which depends on the likelihood of acquisition) or more longitudinal or cross-facility data are compiled to enable a more statistically reliable estimation of the impact. The fact that stochasticity is such an important feature of transmission of HAI-causing pathogens helps to explain why interventions may have different results through time and location. The underlying stochasticity in transmission of HAI-causing pathogens also makes the impact of a change stochastic. In other words, it is difficult to understand the characteristics of a random event with a highly irregular probability distribution by taking only a limited number of observations. For example, for an infection control intervention that is implemented in one or more ICUs, the corresponding impact on acquisition rate may vary across the ICUs or for any single ICU through time. For evaluation purposes, longitudinal data from all the ICUs should be analysed using uncertainty analysis techniques, such as our proposed framework, to understand the probabilistic impact of the implemented intervention.

As our simulations suggest, to overcome the seasonality detection problem, we need to collect a greater amount of data. Our framework presents a mechanism to assess the amount of required data to detect the impact of changes in the environment on the acquisition rates in ICUs. For example, our simulations show when the baseline acquisition rate is lower than 20 cases per 1000 patient-days, the impact of seasonality in admission prevalence on the acquisition rate is more likely to be detected when the seasonality strength is at least 30% (i.e. 30% increase in admission prevalence during the high season). More accurately, our framework can be used to estimate the probability distribution of the potential impact of environmental changes (see figure 6 for an example). Again, here, seasonality is taken as an example measure of a change in the environment; however, we could model the potential impact of any intervention in a healthcare facility and calculate the probability distribution of detection for a given intervention size.

**Figure 7.**
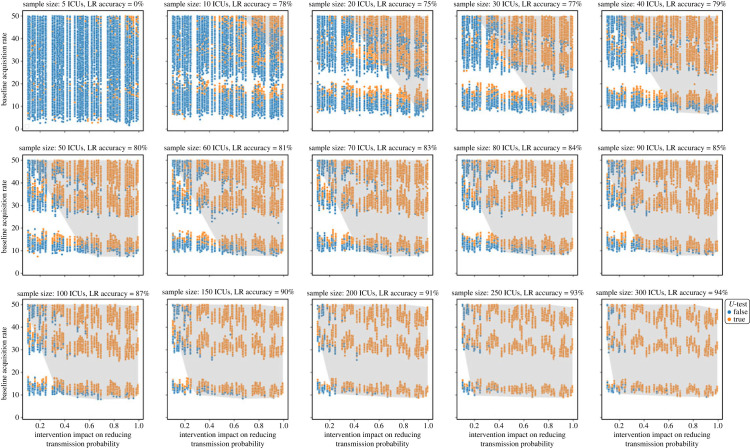
Effect of sample size on the detectability of the impact of an intervention to reduce probability of transmission as a function of baseline acquisition rate (cases per 1000 patient-days) and intervention size. Green circles represent simulations for which the impact of the intervention was detectable using the *U*-test, and the blue ones are simulations in which the intervention impact was undetectable. The shaded area shows the results of the logistic regression predictor (the parameter space corresponding to the ‘true’ intervention impact detection predictor).

**Figure 8.**
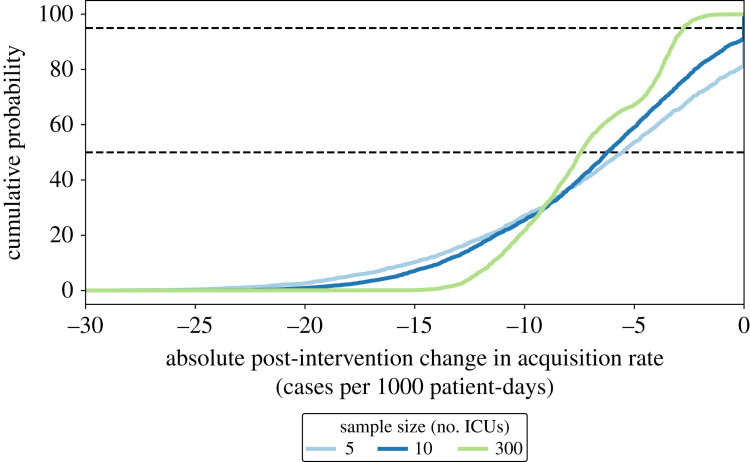
Cumulative probability distribution of the impact of an intervention on acquisition rate for different sample sizes (number of ICUs). The intervention was designed such that it could reduce the probability of transmission during HCW–patient visits by 50% [CI: 5 percent-points].

To demonstrate the application of our methodology to intervention evaluation, we simulated an intervention that affected the mean probability of transmission. For generalizability, we did not implement a specific intervention, rather we changed the mean probability of transmission after a pre-determined intervention time. As the baseline acquisition rate, intervention size, and the number of ICUs in the analysis (sample size) increased, the impact of the interventions became more statistically detectable (figure 7). As an example, for an intervention that could reduce the probability of transmission by 50%, the impact of such an intervention could not be detected with a sample size of 10 ICUs or smaller. With a 20-ICU sample size, the impact of the intervention could only be detected when baseline acquisition rate was at least 30 cases per 1000 patient-days. Increasing the sample size to 50 ICUs could successfully increase our statistical power to detect the impact of the intervention even when acquisition rate was lower than 15 cases per 1000 patient-days. Furthermore, with a 5-ICU sample size, the expected (median or 50-percentile) and 95-percentile impact of the intervention was estimated to be 5.5 and 0.0 cases per 1000 patient-days reduction in acquisition rate, respectively, while increasing the sample size to 300 or more ICUs revealed that such an intervention should be expected to reduce acquisition rate by 7.5 and 2.8 cases per 1000 patient-days, at 50- and 95-percentile, respectively (figure 8).

Understanding the amount of data needed to overcome the stochasticity effect and detect the impact of interventions on lowering the acquisition rate is crucial for deciding what types of interventions to invest in. This is not hospital or ICU independent but depends crucially on the epistemic uncertainty in operations at the healthcare facility. Greater data capture of facility operations, including items such as HCW–patient contact rates, colonization prevalence, and handwashing adherence and effectiveness, can reduce the epistemic uncertainty, which may reduce the data compilation needs. For example, based on the uncertainty in the model and the inherent stochasticity in the system, our simulations suggest that data from at least 50 ICUs would be needed to detect seasonality effects with statistical robustness. However, the ICUs in this case were fairly generic representations of actual ICUs. Though based on data from actual hospitals, there was still a significant amount of uncertainty in many parameters, such as the probability of room and hand colonization and even the rate that nurses visit patients. Development of a more accurate synthetic population would reduce the epistemic uncertainty, and studies that can provide greater understanding of the actual colonization prevalence would likely reduce the required number of ICUs needed to detect a change in the underlying dynamics. Future work will assess the potential of utilizing hospital specific data to better understand the requirements for detecting the impact of interventions.

In conclusion, our findings help explain why certain interventions show inconsistent results through time or across different ICUs. In addition, our framework can be used to provide decision-makers with insights about the minimum required size of interventions given the estimated rate of acquisition in a healthcare facility. The framework can also be used to understand which data elements have the greatest impact on epistemic uncertainty and can be leveraged to reduce the threshold for intervention detection. Furthermore, it can help justify (or invalidate) the application of simpler deterministic macro-scale models, such as compartmental models, to study transmission at the community–hospital interface and evaluate the long-term impact of interventions, given the level of stochasticity in the system. The compartmental susceptible–infected–recovered (SIR) models have been extensively used to study the transmission of infectious diseases. While these models are great tools that are simple to understand and use, they disregard the effects of stochasticity and heterogeneity by assuming deterministic parameters and homogeneous populations. Therefore, they are not suitable for the study of HAI transmission at the facility level due to the underlying high degrees of stochasticity. Our framework can be used to inform the scale (number of ICUs) at which the collective patterns in HAI data converge (e.g. effects of seasonality or impacts of intervention), hence, potentially enabling the application of simpler macro-scale deterministic SIR models.

## Data Availability

Data and relevant code for this research work are stored in GitHub: https://github.com/CDDEP-DC/ABMSeasonality and have been archived within the Zenodo repository: https://zenodo.org/badge/latestdoi/588736534 [39]. Supplementary material is available online [40].
